# Efficacy of Traditional Chinese Medicine in Xerostomia and Quality of Life during Radiotherapy for Head and Neck Cancer: A Prospective Pilot Study

**DOI:** 10.1155/2016/8359251

**Published:** 2016-08-21

**Authors:** Pei-Yu Hsu, Sien-Hung Yang, Ngan-Ming Tsang, Kang-Hsing Fan, Chia-Hsun Hsieh, Jr-Rung Lin, Ji-Hong Hong, Yung-Chang Lin, Hsing-Yu Chen, Cheng-Tao Yang, Ching-Wei Yang, Jiun-Liang Chen

**Affiliations:** ^1^Department of Traditional Chinese Medicine, Division of Chinese Internal Medicine, Taoyuan Chang Gung Memorial Hospital, Taoyuan 33378, Taiwan; ^2^School of Chinese Medicine, Chang Gung University, Taoyuan 33302, Taiwan; ^3^Department of Radiation Oncology, Linkou Chang Gung Memorial Hospital, Taoyuan 33305, Taiwan; ^4^Department of Internal Medicine, Division of Hematology-Oncology, Linkou Chang Gung Memorial Hospital, Taoyuan 33305, Taiwan; ^5^Department of Chemical and Materials Engineering, Chang Gung University, Taoyuan 33302, Taiwan; ^6^Clinical Informatics and Medical Statistics Research Center and Graduate Institute of Clinical Medicine, Chang Gung University, Taoyuan 33302, Taiwan; ^7^Department of Medical Imaging and Radiological Sciences, College of Medicine, Chang Gung University, Taoyuan 33302, Taiwan; ^8^College of Medicine, Chang Gung University, Taoyuan 33302, Taiwan

## Abstract

Xerostomia is one of the most common acute and late complications of radiotherapy for head and neck cancer, and it affects quality of life. We conducted a prospective study to evaluate the efficacy of traditional Chinese medicine (TCM) in toxicities and quality of life during radiotherapy. Head and neck cancer patients who were scheduled for radiotherapy were checked for inclusion/exclusion criteria before enrollment. Patients in the study group (inpatients) were hospitalized in a Chinese medicine ward and received concomitant TCM intervention during radiotherapy, while those in the control group (outpatients) received only conventional cancer treatments at the Western outpatient department. The primary end point was amelioration of postradiotherapy side effects. The secondary end points were quality of life during the cancer therapy and occurrence of adverse events following the TCM treatments. Thirty inpatients and 50 outpatients completed the study. Compared to the control group, those in the TCM group had decreased severity of xerostomia. There was no treatment-related impairment of renal or hepatic function among TCM group. Although better outcomes of social contact, dyspnea, physical and emotional function, and financial problems were found in the TCM group, we need further confirmation about the impact of hospitalization itself on these results.

## 1. Introduction

Head and neck cancer includes tumors of the oral cavity, pharynx, larynx, nasal cavity, paranasal sinuses, thyroid, and salivary glands. Globally, oral and pharyngeal cancer represents the sixth most common cancer worldwide, and Taiwan is one of the highest incidences of oral cancer [1]. According to the Cancer Registry Annual Report in Taiwan, the incidence of oral, oropharyngeal, and hypopharyngeal cancer ranked fourth among all cancers in men in Taiwan, and the death rate also ranked fourth. Besides, head and neck cancer has remained one of the top ten causes of death during recent years.

Common treatments for head and neck cancer include surgery, radiotherapy, and chemotherapy or target therapy. Most patients receive radiotherapy (RT). Common side effects of RT include mucositis, xerostomia, dysphagia, dermatitis, weight loss, and malnutrition. Among these, xerostomia is one of the most common acute and long-term side effects. Xerostomia is manly induced by RT and is dependent on the cumulative radiation doses to the head and neck region [2].

Some patients may not tolerate the side effects of conventional cancer treatment, which may delay or interrupt treatment, and several authors have reported increasing use of complementary and alternative medicine (CAM) among cancer patients [3–5]. In one European study, 22.7% of patients with head and neck cancers used CAM, and the authors found an eightfold increase in the use of herbs after the diagnosis with cancer [3]. Furthermore, there were no treatment delays associated with CAM among head and neck cancer patients [6].

According to the theory of traditional Chinese medicine (TCM), radiation is a type of fire/heat evil that damages yin, manifesting as a lack of fluid and nutrition, and depletes qi, which affects the strength of the constitution. Chemotherapy is also considered to result in lack of qi. Thus, patients who receive cancer treatments may generally have a pattern of lack of both yin and qi. In addition, cancer patients may have varying degrees of psychological problems which is thought to be the syndrome of stagnation of liver qi and blood deficiency by TCM. TCM treatment is based on the principles of nourishing yin, clearing heat, dispersing stagnated liver qi and supplementing blood. However, to date, there have been few evidence-based assessments of the efficacy of TCM treatment for patients with head and neck cancer.

In view of this, we have examined the efficacy of integrated treatment with TCM for head and neck cancer patients for reducing side effects caused by conventional cancer treatments as well as for helping patients to complete their treatments more smoothly and for providing them with improved quality of life.

## 2. Methods

We hypothesized that integrated treatment with TCM for head and neck cancer patients can reduce side effects after RT and provide better quality of life for them. To test the hypotheses, a prospective interventional nonrandomized study of symptom control and quality of life for head and neck cancer patients during RT with or without chemotherapy was conducted from 1 February 2013 to 20 January 2015. The trial registry number is ISRCTN32748291 (http://www.isrctn.com/ISRCTN32748291).

### 2.1. Study Population

Male and female patients aged 20 to 75 years who were first diagnosed with head and neck cancer by Radio-Oncologists or Oncologists at Linkou Chang Gung Memorial Hospital in Taiwan and who were to receive RT with or without chemotherapy were considered for enrollment in this study. Patients in the study group were admitted to the Chinese medicine ward at Taoyuan Chang Gung Memorial Hospital and received integrated TCM treatment from the first week of RT until the end of RT or for at least 6 weeks during RT. The control group received only conventional cancer treatments at the Radio-Oncology or Oncology outpatient department. Patients chose to be in the study group (inpatients) or the control group (outpatients) by self-selection.

The inclusion criteria were normal level of consciousness, normal vital signs (body temperature: 36 to 37.5°C, heart rate: 60 to 100 beats per minute, respiratory rate: <20 per minute, and mean arterial pressure: 70 to 100 mmHg), and Eastern Cooperative Oncology Group (ECOG) performance status of 0 to 2. The exclusion criteria were terminal cancer for which aggressive treatments were not suitable, unstable vital signs, ECOG performance status of ≥3, impaired renal or hepatic function at initial diagnosis (including chronic kidney disease stages III, IV, and V and AST, ALT ≥5 × the upper normal limit), uncontrolled psychiatric problems or altered mental status, and use of other Chinese medicine treatments. All patients were checked for inclusion/exclusion criteria before they could enroll in the study. Inpatients would get partial support of their hospital costs, and outpatients would be provided with tube feeding milk to increase compliance. Recruitment would be stopped ahead of the trial end date if the hospital costs reached the upper limit that our research funding could offer.

### 2.2. Chinese Medicine Interventions

Both groups received conventional cancer treatments including surgery and RT with or without chemotherapy according to their disease status. As noted, patients in the study group were hospitalized in the Chinese medicine ward and received integrated TCM from the first week of RT through the end of RT, or for at least 6 weeks during RT.

According to the precepts of TCM, RT is a kind of fire/heat evil that damages the body's yin and wastes qi. The TCM prescriptions were based on the theory of nourishing yin, clearing heat, and supplementing qi. In addition, cancer patients generally have some level of emotional or sleep disturbance related to the disease itself or to worrying about the diseases. Thus, the TCM we prescribed also addressed the principle of dispersing stagnated liver qi and supplementing the blood to relieve emotional problems and insomnia.

The TCM formulations consisted of concentrated extract powders manufactured by certified pharmaceutical companies, liquid preparations for gargling made from herbal medicines, and topical gels. The medications were prescribed to each inpatient based on symptoms and clinical findings.

Attending physicians from the Chinese Internal Medicine Division of the Department of Traditional Chinese Medicine, Chang Gung Memorial Hospital, Linkou, Taipei, and Taoyuan branch, conducted a consensus conference for standardization of the TCM protocols, and the sample TCM regimens recommended for each side effect are listed in Table 1. The protocols were standardized, but application was tailored to each patient's needs. For example, we used Zeng Ye Decoction (gargle) for mucositis and Angelica Aloe vera gel for dermatitis (topical use). For pharyngitis and/or dry mouth, Qing Yan Li Ge Tang and/or Gan Lu Yin would be prescribed to clear heat and nourish yin. For fatigue and/or poor appetite, we gave inpatients Bu Zhong Yi Qi Tang and/or Xiang Sha Liu Jun Zi Tang to supplement qi. For depression and/or insomnia, we used Jia Wei Xia Yao San and/or Suan Zao Ren Tang to disperse stagnated liver qi and supplement blood. Medications were dispensed to patients in the study group by nurses in the Chinese medicine ward according to physicians' order sheets. All participants were told to avoid other therapies, such as nutritional supplements containing Chinese medicine or acupuncture, in order to minimize confounding factors.

### 2.3. Data Collection

The primary end point was alleviation of side effects after RT. Both subjective and objective changes were evaluated. The European Organization for Research and Treatment of Cancer (EORTC) Quality of Life Questionnaire- (QLQ-) H&N35 had been tested in 12 countries with proven value in the assessment of health-related quality of life (HRQoL) for head and neck cancer patients [7]. We used the EORTC QLQ-H&N35 to evaluate the subjective effects of RT with or without chemotherapy. Patients completed the questionnaire with the help of two trained research assistants once weekly from the second week of RT (visit 2) until the end (visit 7).

The objective effects, including dermatitis, mucositis, xerostomia, and pharyngitis, were assessed according to the Radiation Therapy Oncology Group (RTOG) acute radiation morbidity scoring criteria by well-trained clinicians once weekly since the first week of RT until the end.

The secondary end points were quality of life during cancer treatment, as assessed by the EORTC QLQ-C30 (version 3.0), and changes in renal or hepatic function after TCM. Version 3.0 of the EORTC QLQ-C30 had been confirmed to have improved validity than the previous version and in conjunction with QLQ-H&N35 could be regarded as a reliable and valid instrument for measurement of HRQoL in head and neck cancer patients [7, 8]. Patients completed the questionnaire at week 1 (visit 1), week 2 (visit 2), week 4 (visit 4), and week 7 or at the end of RT (visit 7), and renal and hepatic functions were checked at the beginning and at the end of the TCM treatments.

Patients in the study group completed the above questionnaires and were evaluated for acute radiation toxicities in the Chinese medicine ward and patients in the control group were evaluated at the Radio-Oncology outpatient department.

### 2.4. Statistical Analysis

The trends of change over time in each scale/item of the EORTC QLQ-H&N35 and the EORTC QLQ-C30 were compared between groups by generalized estimating equation (GEE) using SAS software version 9.4. Statistical significance was assumed at *P* < 0.05.

Fisher's exact test (IBM SPSS Statistics 20) was used to compare differences in tumor staging and location, modes of treatment, and adverse events after RT between groups.

### 2.5. Ethical Considerations

This study was approved by the Institutional Review Board of the Chang Gung Memorial Hospital in Taiwan (number 101-2802A3). Written informed consent was obtained from each participant.

## 3. Results

### 3.1. Patient Characteristics

**Figure 1:**
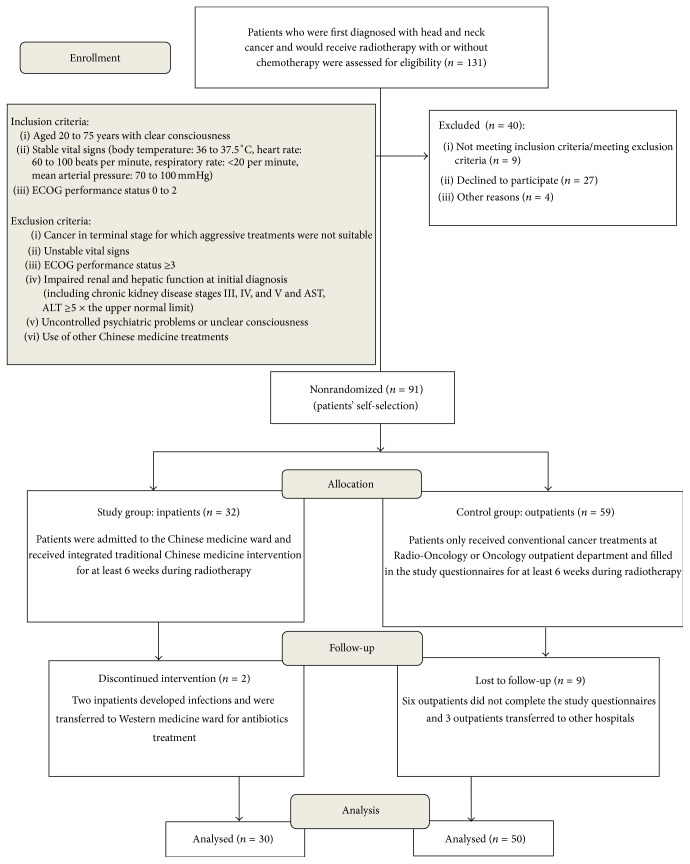
Flow diagram of study enrollment. ECOG, Eastern Cooperative Oncology Group.

From March 2013 to December 2014, 131 head and neck cancer patients at Linkou Chang Gung Memorial Hospital in Taiwan were assessed for eligibility. Forty patients were excluded from the study. Among them, 9 patients did not meet the inclusion criteria and/or met the exclusion criteria, 27 patients declined to participate, and 4 patients decided to transfer to other hospitals. A total of 91 head and neck cancer patients were enrolled in the study. Thirty-two patients chose to participate as inpatients in the Chinese medicine ward (study group) and 59 chose conventional treatment as outpatients at the Radio-Oncology or Oncology outpatient department (control group). Patients participated in the study from the first week of RT until the end of RT and/or for at least 6 weeks during RT. All patients received RT with or without chemotherapy at Linkou Chang Gung Memorial Hospital. A total of 30 inpatients and 50 outpatients completed the study; 2 inpatients and 9 outpatients dropped out of the study. The 2 inpatients did not complete the study because they developed infections and were transferred to Western medicine ward for antibiotics treatment. Six outpatients did not complete the study questionnaires and the other 3 outpatients transferred to other hospitals (Figure 1).

The tumors were classified as nasopharyngeal carcinoma (NPC), cancers of the oral cavity and pharynx, and other head and neck cancers, including one thyroid cancer in the inpatient group and one laryngeal cancer in the outpatient group. Baseline characteristics of the participants, including parameters such as gender, age, height, body weight, body mass index (BMI), tumor stage, location, modes of treatment, and mean dose of RT, are shown in Table 2. Other than dose of RT (70.47 ± 3.44 Gy, inpatients, versus 69.20 ± 2.14 Gy, outpatients, *P* = 0.045), there were no significant differences between groups. Patients generally received RT with the dose of 2 Gy per day on weekdays and the duration of around 7 weeks. Except for 4 inpatients and 3 outpatients who did not receive chemotherapy, most patients received RT with chemotherapy.

### 3.2. Quality of Life Questionnaire, Head and Neck Module (EORTC QLQ-H&N35)

The EORTC QLQ-H&N35 assesses impacts on quality of life that are specific to head and neck cancer. It includes seven multi-item scales for assessment of pain, swallowing, sensation, speech, social eating, social contact, and sexuality. There are also 11 single-item scores for dentition, mouth opening, dry mouth, sticky saliva, coughing, ill feeling, analgesic requirement, use of nutritional supplements, feeding tube requirement, weight loss, and weight gain. For all items and scales, higher scores indicate greater severity of adverse effects [9].

**Figure 2:**
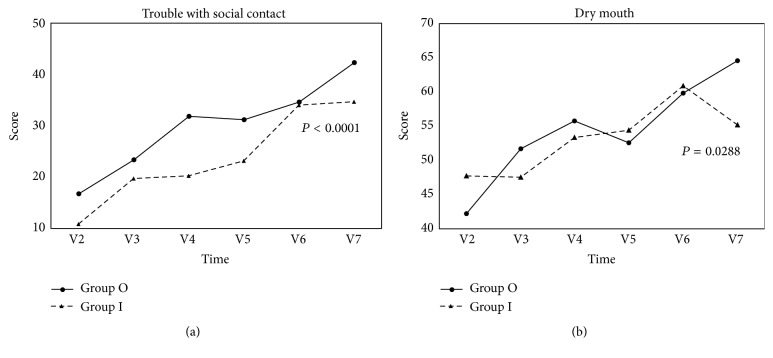
Results of EORTC QLQ-H&N35. The trends of change over time in each scale/item for 30 patients in the study group and 50 patients in the control group were compared by generalized estimating equation (GEE). The abscissas represent time (week) of visit; the ordinates represent mean score for each scale/item. Statistical significance was assumed at *P* < 0.05. (a) Patients in the study group had less deterioration of social contact (*P* < 0.0001) and (b) decreased severity of dry mouth (*P* = 0.0288) over time compared with the patients in the control group. Group O, outpatient (control) group; group I, inpatient (TCM) group; V2, visit 2; V3, visit 3; V4, visit 4; V5, visit 5; V6, visit 6; and V7, visit 7.

We compared the trends of change for each item or scale during the treatment course (from visit 2 until visit 7) between the study group (*n* = 30) and the control group (*n* = 50). GEE was used for the statistical analysis because some data were missing. Patients in the study group had less deterioration of social contact (*P* < 0.0001) and decreased severity of dry mouth (*P* = 0.0288) over time compared with patients in the control group (Figure 2).

### 3.3. Acute Post-RT Toxicity

The severity of RT-induced dermatitis, mucositis, xerostomia, and pharyngitis was graded according to the RTOG acute radiation morbidity scoring criteria. Generally, the RTOG toxicity scores measured for each patient at visit 1 in both groups were 0 or 1, but there were exceptions for 2 inpatients and 1 outpatient who had grade 2 xerostomia, and 1 inpatient had grade 2 pharyngitis at visit 1. Thus, to minimize confounding factors, patients whose RTOG score was not grading as 0 or 1 at visit 1 and those with missing data were excluded from the analysis. The number of patients with no or mild changes at visit 1 (grade 0 or grade 1 at week 1) was used as the baseline for comparison. Severity of side effects was assessed at visit 4 and visit 7. Changes graded as 0 and 1 at visits 4 and 7 were regarded as no/mild change from baseline and those ranked grade 2 or grade 3 were defined as moderate/severe change compared with baseline. There was no significant difference in radiation doses between groups for patients involved in the analysis. There was an inconsistency of patient numbers in the assessment of mucositis because of missing data (Table 3).

The TCM group had less severe xerostomia at visits 4 and 7 (lower proportion of patients with moderate/severe change from baseline, *P* = 0.031 and 0.0495, visits 4 and 7, resp.) compared with the control group (Table 3). There was also a trend toward decreased severity of pharyngitis for patients receiving TCM during RT, although the result did not reach significance (Table 3).

### 3.4. Quality of Life Questionnaire, Generic (EORTC QLQ-C30)

The QLQ-C30 is composed of both multi-item scales and single-item measures of cancer-related impacts on quality of life. These include five functional scales (physical functioning, role functioning, emotional functioning, cognitive functioning, and social functioning), three symptom scales (fatigue, nausea and vomiting, and pain), a global health status scale, and six single items (dyspnea, insomnia, appetite loss, constipation, diarrhea, and financial difficulties). A high score represents a higher response level. Thus, a high score for a functional scale represents a high/healthy level of functioning and a high score for the global health status represents a high quality of life, but a high score for a symptom scale/item represents high severity of the symptom [9].

Four inpatients and 3 outpatients who did not receive chemotherapy were excluded from the analysis because patients who do not receive chemotherapy are generally thought to have better quality of life, and many items in this questionnaire, such as those related to gastrointestinal toxicities, are more likely related to chemotherapy. Thus, 26 patients in the TCM group and 47 in the control group were included in this portion of the analysis. We compared the trends of change between groups for each scale/item at weeks 1, 2, 4, and 7 of the treatment course.

**Figure 3:**
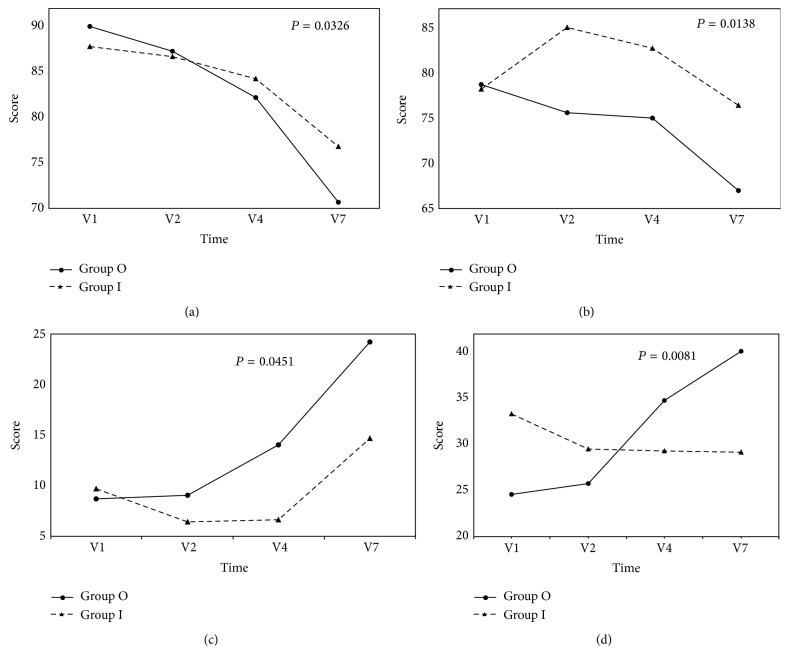
Results of EORTC QLQ-C30. Twenty-six patients in the TCM group (inpatients) and 47 in the control group (outpatients) were compared; 4 inpatients and 3 outpatients who did not receive chemotherapy were excluded from the analysis. The abscissas represent time of visit; the ordinates represent mean score for each scale/item. The trends of change over time in each scale/item were compared between groups by generalized estimating equation (GEE). Statistical significance was assumed at *P* < 0.05. The results showed better physical functioning (a) (*P* = 0.0326) and emotional functioning (b) (*P* = 0.0138) as well as lower incidence of dyspnea (c) (*P* = 0.0451) and fewer financial difficulties (d) (*P* = 0.0081) for inpatients compared with outpatients. Group O, outpatient (control) group; group I, inpatient (TCM) group; V1, visit 1; V2, visit 2; V4, visit 4; and V7, visit 7.

The results of the QLQ-C30 indicated that patients who received TCM maintained better physical and emotional function (*P* = 0.0326 and 0.0138, resp.) compared to the control group. The TCM group also had a lower incidence of dyspnea (*P* = 0.0451) and fewer financial difficulties (*P* = 0.0081) (Figure 3).

### 3.5. Renal and Hepatic Function

There was no renal or hepatic function impairment detected after TCM treatment in the study group.

## 4. Discussion

In recent years, the use of CAM in combination with conventional cancer therapy has become more widespread, and a systematic review has shown that Chinese herbal medicines, including Astragalus, Turmeric, and Ginseng, can enhance the efficacy of chemotherapy and radiotherapy while reducing posttherapy side effects via immune modulation and anti-inflammatory, anticancer, and antioxidant mechanisms [10, 11]. TCM has also been shown to protect the liver during chemotherapy [12].

In a study of patients with NPC, those who received conventional anticancer therapies combined with TCM had increased survival rates, enhanced tumor response, improved Karnofsky performance status, reduced rates of adverse effects, and augmented immunostimulation [13]. Our previous study showed that head and neck cancer patients who received TCM treatment along with conventional therapies experienced less weight loss during RT, and this effect was enhanced when patients received TCM for a longer period of time [14].

Improved treatment techniques for head and neck cancers have led to improved survival rates for patients. However, acute treatment-related toxicities are common and develop during or shortly after the completion of treatment. Common acute toxicities such as salivary gland damage or xerostomia, mucositis, dermatitis, dysgeusia, orofacial pain, weight loss, and malnutrition are bothersome and at worst can lead to serious complications or interruption of treatment. HRQoL generally declines during cancer treatment but will gradually recover to baseline levels. Combined chemoradiotherapy shows a trend toward greater declines in HRQoL compared with RT alone [15].

Oral complications such as dry mouth, change in taste, and dysphagia are the main problems after RT for patients with head and neck cancers, and these adverse effects often lead to diminished quality of life [16]. Decreased saliva production or xerostomia becomes evident within one to two weeks after the initiation of RT. Permanent reduction of saliva production can occur with cumulative radiation doses to the parotid gland of 10 to 15 Gray. Pronounced reduction of gland function (>75%) usually occurs at >40 Gray [17]. Previous reports have shown that xerostomia-related quality of life worsened at the end of RT and persisted for 1 to 2 years [18, 19]. Xerostomia can lead to mucosal ulceration and pain, difficulty in swallowing, altered taste sensation, increased risk of oral infections, and dental caries [20, 21]. Xerostomia, swallowing difficulty, and poor dentition may result in eating problems followed by nutritional deficiencies and weight loss [22, 23].

Current Western medications for xerostomia include amifostine, pilocarpine, and bethanechol. However, the use of amifostine is limited by cost, inconvenience of intravenous infusion, and side effects including hypotension and nausea and vomiting. Subcutaneous amifostine was thought to be more convenient and less toxic, but efficacy and patient compliance have been no better when compared with intravenous use [24]. Pilocarpine and bethanechol both have muscarinic side effects, including sweating, nausea, dizziness, urinary frequency, and asthenia [25]. Other treatments such as acupuncture, electrical nerve stimulation, hyperbaric oxygen therapy, salivary substitutes, or salivary stimulants such as chewing gum, ascorbic acid (vitamin C), and malic acid might also be useful, but the effects are controversial [26–28].

According to the theory of TCM, radiation is a type of fire/heat evil that damages yin, manifesting as a lack of fluid and nutrition, and depletes qi, which affects the strength of the constitution. Thus, we prescribed TCM according to the principle of nourishing yin and clearing heat to treat mucositis, pharyngitis, and dry mouth and of supplementing qi to treat fatigue and poor appetite. Our results showed that, for patients who received complementary TCM, both the subjective perception of dry mouth and the objective findings recorded by clinicians were less severe than in the control group. There was also a trend toward decreased severity of pharyngitis for patients receiving TCM, although the result did not reach significance. There were no recognizable differences for fatigue and appetite between the groups, but the TCM patients had better physical function and less dyspnea, probably related to supplementing qi.

Other treatment goals of TCM included dispersing stagnated liver qi and supplementing the blood to relieve emotional depression and insomnia. Previous studies have shown that psychosocial problems such as depression, anxiety, and social phobia are important factors that can decrease quality of life during treatment [29] and have confirmed that depression decreases quality of life [30, 31]. We found that patients who received TCM had better emotional function and less difficulty with social contact than patients who received conventional treatments alone. The relative better physical conditions might contribute to less financial strain for the inpatients.

While our study had proved the hypotheses that integrated treatment with TCM for head and neck cancer patients can reduce xerostomia after RT, there were some confounding factors about quality of life related outcomes. First, we do not know what impact the prolonged hospitalization itself had for the patients who received TCM. Some of the patients who lived far from our center felt that hospitalization was more convenient because they would otherwise have had to make long commutes for RT. Patients might be benefited from more rest during hospitalization as well as from TCM treatment by supplementing qi to improve physical function and dyspnea. Besides, some patients felt that they had better emotional support from doctors and nurses during their hospital stay than they may otherwise have had. However, some patients felt bored and found that being at the hospital was inconvenient and that they missed their routine meals and family support while being at the hospital. These made the results of emotional and social function confusing. Second, human papillomavirus (HPV) status in patients with head and neck cancer is also a confounding factor because this subset is typically healthier and has better prognosis [32–34]. Third, we could not know whether the reduced financial strain for inpatients was due to better health status or because they received insurance benefits during hospitalization.

There are some other limitations to this study. First, randomization was difficult because hospitalization in the Chinese medicine ward is self-paid. Other characteristics such as patients who were more traditional in their beliefs or more anxious or lived alone might have influenced the decision to be hospitalized. Second, TCM is characteristically an individualized treatment based on pattern identification (i.e., bian zheng lun zhi), and although the formulations were standardized, the prescriptions were not. Third, HPV status of each patient in this study was not routinely checked because the majority of our participants did not have oropharyngeal cancer, which was shown to have higher prevalence of HPV positive status than nonoropharyngeal head and neck cancer [35, 36]. Fourth, we could not follow our patients for a prolonged period after discharge. Thus, it was not possible to determine if TCM remained effective for long-term use. However, this study has provided an evidence-based treatment strategy for xerostomia during RT.

## 5. Conclusion

Our study has demonstrated that TCM treatment during RT for head and neck cancer could reduce the severity of xerostomia. There was no impairment of renal or hepatic function detected after TCM treatment in the study group. As for the aspects of less difficulty with social interactions, reduced occurrence of dyspnea and financial problems, and better physical and emotional function, we cannot distinguish whether they benefited from TCM treatment, hospitalization, or both. Further studies including evaluating the effect of the hospitalization itself, collecting HPV status, and addressing long-term follow up and prognosis for these patients may be conducted.

## Figures and Tables

**Table 1:** Recommended TCM treatments.

Symptom	Prescription	Example doses	Remarks
Mucositis	Zeng Ye Decoction^a^		Gargle
Pharyngitis	Qing Yan Li Ge Tang	1 to 2 g TID	
Dry mouth	Gan Lu Yin	2 to 4 g TID	
Fatigue	Bu Zhong Yi Qi Tang	2 to 3 g TID	
Poor appetite	Xiang Sha Liu Jun Zi Tang	2 to 3 g TID	
Dermatitis	Angelica Aloe vera gel^b^		Topical use
Emotional depression	Jia Wei Xia Yao San	2 to 3 g TID	
Insomnia	Suan Zao Ren Tang	3 to 4 g HS	

TCM, traditional Chinese medicine.

Except for Zeng Ye Decoction and Angelica Aloe vera gel, all the other oral Chinese medicines are concentrated extract powders that are made by the Sun Ten, Sheng Chang, Chuang Song Zong, and Ko Da pharmaceutical companies in Taiwan. The powder is generally given 3 times a day after meals and/or before sleep.

^a^Zeng Ye Decoction is made from herbal extracts of *Rehmannia glutinosa*, *Scrophularia ningpoensis*, and *Ophiopogon japonicus* to form liquid preparation for gargling.

^b^Angelica aloe vera gel is made by the Formosa Biomedical Technology Corporation in Taiwan. The gel is usually applied topically 2 or 3 times a day.

**Table 2:** Patient characteristics.

Variable	Inpatients (*n* = 30)	Outpatients (*n* = 50)	*P*
Gender (male : female)	23 : 7	44 : 6	0.22
Age in years (mean ± SD)	49.63 ± 10.17	47.68 ± 7.91	0.34
Height in meters (mean ± SD)	1.67 ± 0.08	1.67 ± 0.06	0.81
Body weight in kg (mean ± SD)	68.00 ± 13.90	68.52 ± 12.53	0.86
BMI in kg/m^2^ (mean ± SD)	24.34 ± 4.38	24.45 ± 4.22	0.91
TNM stage (AJCC, 2009); *n*			0.91
Stage I	2	5	
Stage II	6	8	
Stage III	8	12	
Stage IV	14	25	
Tumor location (*n*)			0.75
NPC	15	22	
Oral cavity	7	17	
Pharynx	7	10	
Others	1	1	
Modes of treatment (*n*)			0.35
Surgery + radiotherapy + chemotherapy	6	15	
Radiotherapy + chemotherapy	20	32	
Surgery + radiotherapy	2	2	
Radiotherapy	2	1	
Dose of radiotherapy in Gray (mean ± SD)	70.47 ± 3.44	69.20 ± 2.14	0.045^a^

AJCC, American Joint Committee on Cancer; NPC, nasopharyngeal carcinoma; BMI, body mass index.

Age, height, body weight, BMI, and dose are presented as means ± standard deviation.

Significant difference between the 2 groups was found only for dose of radiotherapy (inpatients > outpatients, *P* = 0.045).

^a^ *P* < 0.05.

**Table 3:** Acute radiotherapy-induced toxicity.

Symptom	Change from baseline	Visit 4					Visit 7					Total RT dose (Gray, mean ± SD)		
		Inpatients		Outpatients		*P*	Inpatients		Outpatients		*P*	Inpatients	Outpatients	*P*
		*n*	(%)	*n*	(%)		*n*	(%)	*n*	(%)				
Dermatitis	No/mild change	29	(96.7)	37	(80.4)	**0.078**	15	(50)	16	(34.8)	**0.235**	70.47 ± 3.44	69.30 ± 2.12	**0.105**
	Moderate/severe change	1	(3.3)	9	(19.6)		15	(50)	30	(65.2)				

	*Total*	**30**		**46**			**30**		**46**					

Mucositis	No/mild change	19	(65.5)	25	(59.5)	**0.63**	12	(41.4)	16	(37.2)	**0.807**	70.48 ± 3.50	69.21 ± 2.14	**0.087**
	Moderate/severe change	10	(34.5)	17	(40.5)		17	(58.6)	27	(62.8)				

	*Total*	**29**		**42**			**29**		**43**					

Xerostomia	No/mild change	12	(44.4)	8	(19)	0.031^**a**^	8	(29.6)	4	(9.5)	0.0495^**a**^	70.52 ± 3.63	69.19 ± 2.17	**0.095**
	Moderate/severe change	15	(55.6)	34	(81)		19	(70.4)	38	(90.5)				

	*Total*	**27**		**42**			**27**		**42**					

Pharyngitis	No/mild change	17	(60.7)	15	(34.9)	**0.0504**	8	(28.6)	7	(16.3)	**0.245**	70.50 ± 3.56	69.21 ± 2.14	**0.093**
	Moderate/severe change	11	(39.3)	28	(65.1)		20	(71.4)	36	(83.7)				

	*Total*	**28**		**43**			**28**		**43**					

Radiation toxicities were measured according to the RTOG acute radiation morbidity scoring criteria. Fisher's exact test was used to compare differences for distribution of severity for each post-RT finding between groups at visit 4 and visit 7. The number of patients with no or mild changes during the first week of RT (grade 0/1 at visit 1) was selected as the baseline for comparison. Grade 0/1 indicates no/mild change and grade 2/3 indicates moderate/severe change at visit 4 and visit 7 compared with baseline. There was no significant difference in radiation doses between groups for patients involved in the analysis. There was an inconsistency of patient numbers in the assessment of mucositis because of missing data. Inpatients (TCM group) had a lower proportion of moderate/severe change in severity of xerostomia compared with outpatients (control group) (*P* = 0.031 and 0.0495, resp.).

^a^ *P* < 0.05.
